# First person – Hui-Ying Tsai and Shih-Cheng Wu

**DOI:** 10.1242/dmm.046755

**Published:** 2020-08-27

## Abstract

First Person is a series of interviews with the first authors of a selection of papers published in Disease Models & Mechanisms, helping early-career researchers promote themselves alongside their papers. Hui-Ying Tsai and Shih-Cheng Wu are co-first authors on ‘Loss of the *Drosophila* branched-chain α-ketoacid dehydrogenase complex results in neuronal dysfunction’, published in DMM. Hui-Ying is a research assistant in the lab of Chun-Hong Chen at National Health Research Institutes, Zhunan, Taiwan. Her research interest is modeling the human neurological disease maple syrup urine disease in *Drosophila*, assessing behavior as well as brain damage. Shih-Cheng is a postdoc in the same lab, with interests in modeling human disease and immunometabolism.


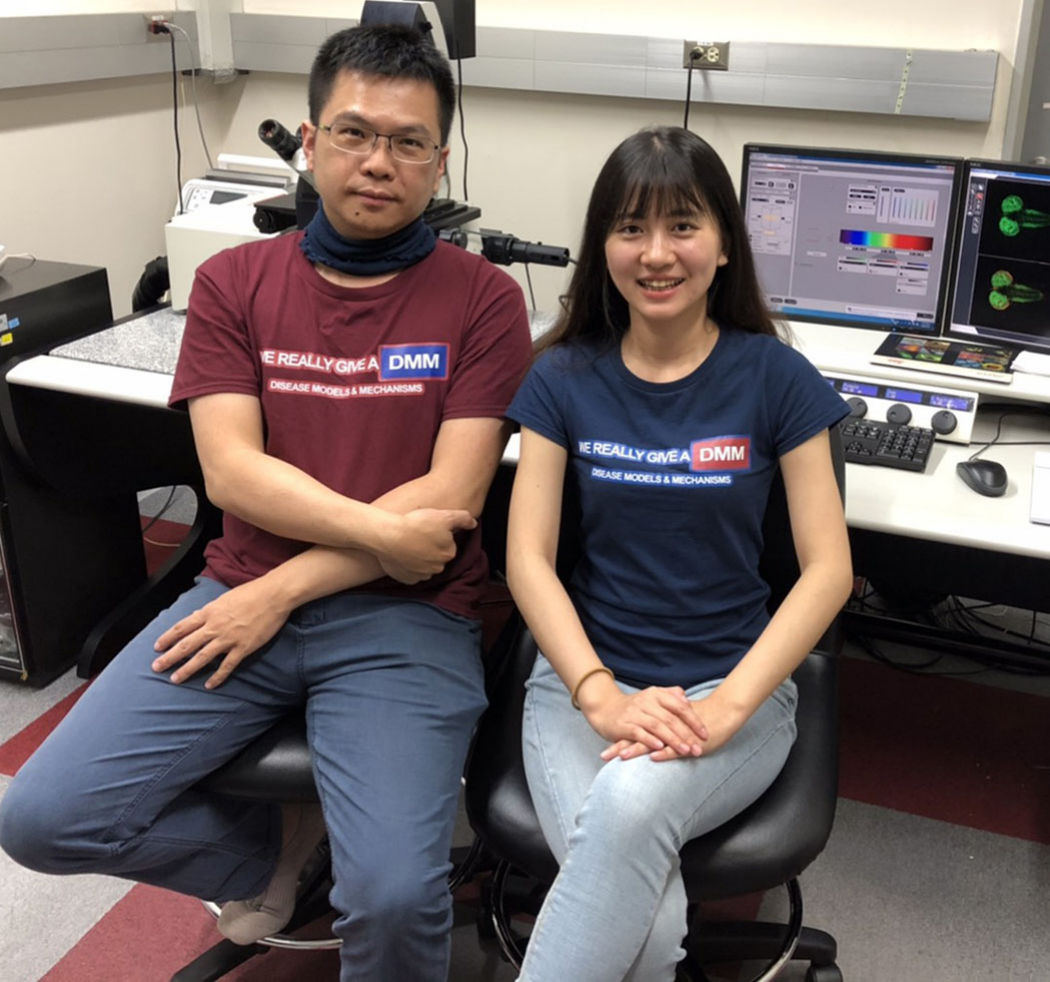


Hui-Ying Tsai (right) and Shih-Cheng Wu (left)

**How would you explain the main findings of your paper to non-scientific family and friends?**

H-YT & S-CW: Amino acid metabolism is responsible for the body's systemic homeostasis, which is dysregulated inherently in maple syrup urine disease (MSUD), which is attributed to abnormally excessive levels of branched-chain amino acids (BCAAs). The lack of effective therapeutics for this disease has long been a concern. In our study, we developed a system using *Drosophila* with BCAA-catabolizing deficiency, potentially providing the ability to elucidate the pathogenesis of MSUD and aid the development of therapeutic methodology.

**What are the potential implications of these results for your field of research?**

H-YT: As there is an urgent requirement to develop an effective drug for MSUD, the mechanism underlying MSUD needs to be further studied. We established a *Drosophila* model to provide a novel and convenient platform to investigate MSUD. In addition to recapitulating clinical manifestations, we showed that oxidative stress plays a potential role in modulating brain apoptosis in the *dDBT* mutant, and provide *in vivo* evidence that apoptosis and reactive oxygen species (ROS) are risk factors leading to neurological illness in our *Drosophila* model of MSUD.

S-CW: Our study presents a newly available approach to model MSUD, and this new *Drosophila* model, along with other MSUD models, could accelerate the development of therapeutics. Moreover, our study of the BCAA-catabolizing mutant unveils the potential to elucidate the interaction of energetic status with various diseases through the *Drosophila* system with convenient genetics.

**What are the main advantages and drawbacks of the model system you have used as it relates to the disease you are investigating?**

H-YT & S-CW: Owing to the advantages of the *Drosophila* system, such as a shorter lifecycle, evolutionary physiology and easier genetic manipulability, the model of *Drosophila* with BCAA-catabolizing deficiency could provide an *in vivo* high-throughput platform for drug screening of MSUD, as an *in vitro* cell-based strategy is limited by the absence of the organismal complexity of an animal system. Since the residual 0-5% BCAA-catabolized activity remains in the most severely affected MSUD patients, in this study, the drawback of the null *Drosophila* mutant system we created was that it may not recapitulate completely all events related to the clinical features of the disease.

**What has surprised you the most while conducting your research?**

H-YT: The surprising thing for me was to verify that *Drosophila* is versatile, and that we can conduct various experiments to mimic human disease in this small animal.

S-CW: Studying the basic research is always the fundamental step towards potentially alleviating, improving or curing human diseases. I am always excited about this process. What surprised me the most was that a diabetic drug, Metformin, may be a candidate medication for MSUD, although preclinical and clinical examination still need to be performed.

**Describe what you think is the most significant challenge impacting your research at this time and how will this be addressed over the next 10 years?**

H-YT: The challenge in studying MSUD is that this is a rare disease without abundant research data and with poorly understood mechanisms. As the *Drosophila* model has now been established, enabling different aspects to be unveiled, more genetic tools and molecular approaches could be valuably manipulated to better understand this disease.

S-CW: A number of model systems have been developed for studying human disease but no specific model is perfectly suitable for recapitulating the features of human diseases. Therefore, ultimately, whether the outcome from our work significantly translates into the aspects of the complicated human system is always a concern. To address this challenge, research achievements from various models might be effective and needed in the future.

**Figure:**
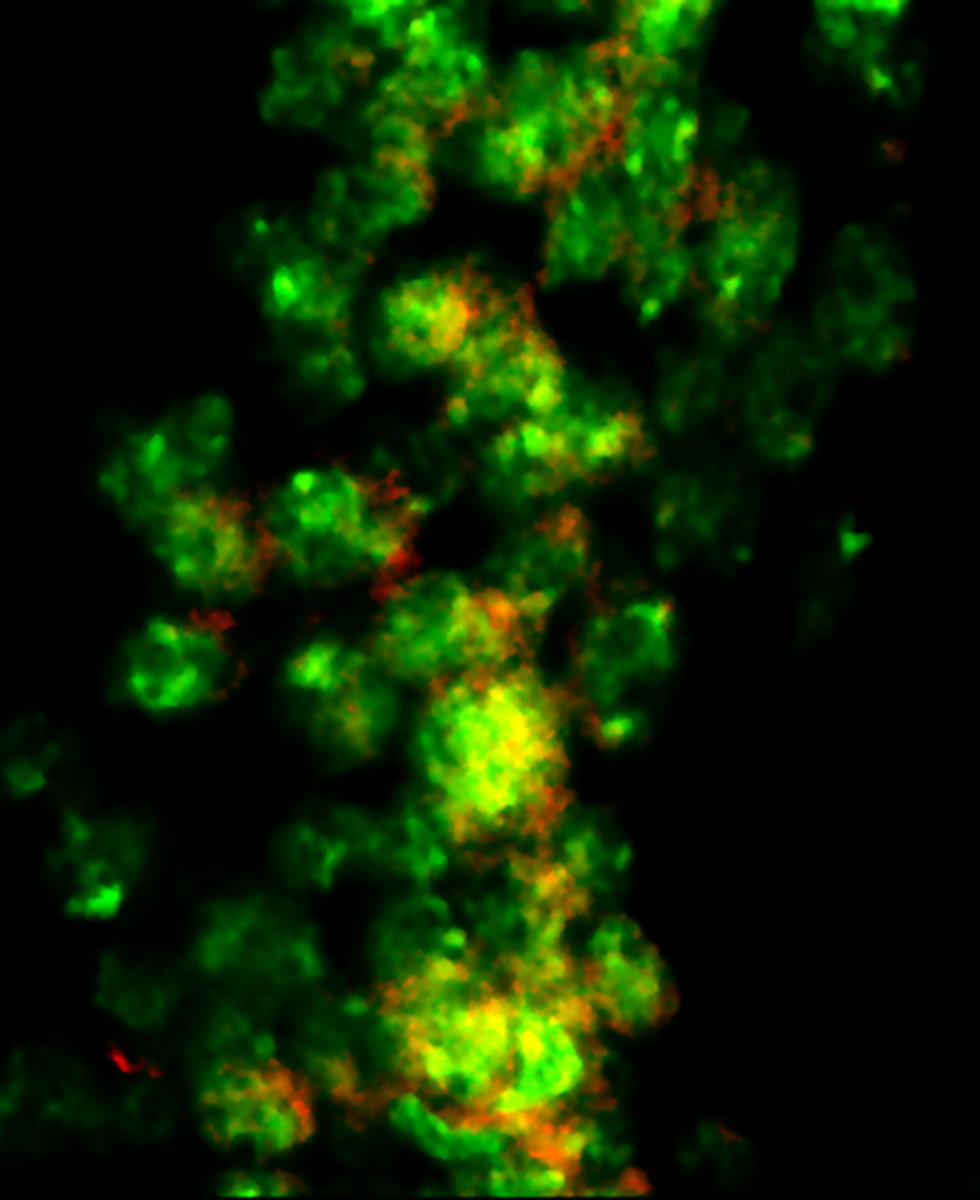
Neuronal apoptosis (red) in pupal brain tissues (green) of *Drosophila* with *dDBT* deficiency.

**What changes do you think could improve the professional lives of early-career scientists?**

H-YT: I believe that it is important to always keep the mind switched on and to never give up on solving the mysteries among your research. In my opinion, keeping reading the references with specific or multidisciplinary research is a good way to achieve this.

S-CW: I personally believe that having scientific and logical thinking is an important asset for scientists when pursuing research work. Endeavor and enthusiasm to create opportunities to obtain funding and scientific collaborations will also be helpful in moving forward as a successful early-career scientist.

**What's next for you?**

H-YT: As ROS stress was found to have a detrimental role in *dDBT* deficiency, the next goal is trying to understand the pathological mechanisms in as much detail as possible, which may help drug discovery for MSUD.

S-CW: The next stage regarding this study of the *Drosophila* model system is to unveil the mechanisms underlying the dysregulated BCAA accumulation-caused illness. I am also looking forward to directing other intriguing research projects in the future.
